# 

**Published:** 1869-04-15

**Authors:** 


VOL. XXVI. NUMBER 8.

### THE CHICAGO Medical Journal.

PUBLISHED SEMI-MONTHLY.

J. ADAMS ALLEN, M. D., LL. D.,
*EDITOR.*

WALTER HAY, A. M., M. D.,

*ASSOCIATE EDITOR.*

APRIL 15, 1869.

CHICAGO:
HORTON & LEONARD, STEAM BOOK AND JOB PRINTERS,
104 & 106 Randolph Street.

